# Consumers' Contribution to Health Research: Australian Research Organisations' Perspectives

**DOI:** 10.1111/hex.70258

**Published:** 2025-04-28

**Authors:** Mingming Zhou, Julia Dray, Anne Parkinson, Alice Richardson, Lucy Clynes, Jane Desborough

**Affiliations:** ^1^ Department of Health Economics, Wellbeing and Society, National Centre for Epidemiology and Population Health Australian National University Canberra Australia; ^2^ Graduate School of Health, Faculty of Health The University of Technology Sydney Sydney Australia; ^3^ Statistical Support Network Australian National University Canberra Australia; ^4^ Research Australia Canberra Australia

**Keywords:** compensation, consumer engagement, consumer partnership, health research organisation, reimbursement, remuneration

## Abstract

**Background:**

Despite growing recognition of the importance of consumer engagement in advancing consumer‐oriented and relevant research, many researchers experience challenges in appropriately acknowledging consumers' contributions to the research process. A pivotal aspect of this challenge relates to the financial remuneration offered to consumers in recognition of their contributions to research in terms of time, skills and expertise. This may be due to a lack and inconsistency of reported practice and guidance regarding remuneration. We sought to explore current practices for recognising consumers' contribution to health research and to understand health organisations' experiences and perspectives regarding this.

**Methods:**

A working group convened by Research Australia comprised of two academics, one PhD candidate and eight health research organisation representatives, including consumer‐led organisations, was established to develop a survey to elucidate current consumer remuneration practices in Australia. Drawing on existing consumer engagement literature, the draft survey questions were constructed and finalised following review, discussion and reaching consensus among the working group. The survey was distributed online to 503 research organisation participants across Australia from December 2023 to February 2024. Quantitative data were analysed using descriptive statistics, and qualitative data were analysed using content analysis methods.

**Results/Findings:**

124 completed surveys were returned (25% response rate). 92% of participants supported recognition of consumer contributions to health research. Of this, 56% provided financial remuneration, while 36% provided non‐financial forms of recognition, such as training and acknowledgement of academic outputs. However, recognition methods used in practice differed, and rates of financial remuneration varied across different levels of engagement. The need for national guidelines in consumer remuneration practice was expressed by 93% of participants.

**Implications/Key Message:**

These survey findings support an imperative to establish national recommendations for consumer remuneration, tailored to the needs of different organisations and contexts in Australia.

**Patient and Public Contribution:**

A working group formed with eight member organisations from Research Australia, including consumer‐led organisations, contributed to the survey development and interpretation of the qualitative findings by reflecting on the themes developed.

## 1 Introduction

Consumers, defined as individuals with lived experience of specific health conditions, offer valuable experiential insights and can contribute to the design, conduct and dissemination of research projects [1, 2]. Despite the growing recognition of the importance of consumer engagement in advancing patient‐oriented research and research quality, researchers face substantial challenges in effectively engaging a diverse spectrum of consumers and appropriately acknowledging their contributions to the research process [3, 4, 5]. Some of these challenges include uncertainty about appropriate recognition methods, inadequate compensation, time and financial constraints, and limited guidance or infrastructure for acknowledging consumers' contributions [3, 6, 7, 8]. These difficulties are further compounded by institutional inflexibility and a lack of policy support [7].

A pivotal aspect of these challenges relates to the issue of remuneration—the financial compensation offered to consumers in recognition of their contributions in terms of time, skills and expertise to research projects [7, 9]. Remuneration can take various forms, including direct compensation and reimbursement for out‐of‐pocket expenses such as travel, parking, meals and childcare [3, 10, 11, 12]. Offering remuneration may allow consumer partners from different socioeconomic population groups to be engaged in research and thus encourage the inclusion of diverse perspectives [10].

To support researchers in engaging consumers in research, various institutions have developed frameworks and guidelines aimed at addressing the intricate issue of remuneration [3]. Examples of these institutions include the National Institute for Health Research (NIHR) from the United Kingdom [13], the Patient‐Centred Outcomes Research Institute (PCORI) from the United States [14], Strategy for Patient‐Oriented Research (SPOR) from the Canadian Institutes for Health Research (CIHR) [15] and the Belgian Health Care Knowledge Centre [16]. Notably, from this latest scoping review, which collected available policies and guidelines for patient partner compensation, no document providing national guidelines was retrieved from Australia [16].

Health consumer organisations within the Australian states and territories, including New South Wales (NSW) [17], Queensland [18], the Australian Capital Territory (ACT) [19], Western Australia (WA) [20] and South Australia (SA) [21], have developed their own remuneration policies for consumer engagement; however, there is variability in rates and types of remuneration in the different jurisdictions across Australia. Little is known regarding how research organisations in Australia recognise consumers' contributions, either financially or non‐financially. Similarly, health organisations' attitudes and perceptions regarding remuneration, including targets (eligible receivers of remuneration) and methods in current practice, remain unclear. Whether it is necessary to build a national consensus on remuneration rates to meet the needs of research practices remains unanswered. Given this significant knowledge gap, we sought to explore the perspectives and practices of Australian health research organisations regarding recognition, including financial remuneration, of consumers' involvement in research.

## 2 Methods

### 2.1 Survey Development and Administration

We developed a survey with a working group formed in collaboration with Research Australia (an organisation that advocates for the health and medical research pipeline). The members of the working group included authors (M.Z., J.D., A.P. and L.C.) and representatives of eight member organisations from Research Australia. The working group met online every 3 months over a period of 1 year to develop and refine the survey, reflect on the results and draft a report of the findings, including the potential to inform the development of national guidelines. The purpose of the survey was to identify how health organisations, including for‐profit and not‐for‐profit, currently recognise consumers for their contributions to research and to understand the experiences and attitudes of health organisation members regarding financially remunerating consumers in health research.

(a) We conducted a rapid scoping review of existing remuneration practices in both Australian and international contexts, followed by consultation and co‐design of a survey with the working group.

(b) The survey was pilot tested with three lived‐experienced researchers who have both personal experience of chronic conditions and extensive experience with the engagement of consumers in health research. Pilot testing helped ensure the survey was accessible, comprehensive and captured the type of experiences we sought.

(c) Data collection: The finalised anonymous survey was distributed to all member organisations of Research Australia (approximately 160). It was administered via Qualtrics (an online survey tool) in English and available online only with no paper‐based option. To enhance participation, we implemented several strategies. First, we sent three rounds of email invitations in December 2023 and January 2024. Additionally, we kept the survey open for 3 months, closing on 29 February 2024, to allow ample time for responses.

We received ethical approval to distribute the survey from the Australian National University Human Research Ethics Committee (Protocol 2023/1263). All participants provided informed consent before completing the survey.

### 2.2 Survey Contents

The survey (Appendix 1) consisted of 67 multi‐choice and open‐text questions and was comprised of five parts: (1) Basic information about a participating organisation, (2) Forms of recognition of consumer engagement, (3) Forms of financial remuneration and reimbursement, (4) Remuneration processes and (5) Perspectives about remuneration guidelines. Part 1 collected information about the type (for‐profit or not‐for‐profit) and size of organisations (large, medium or small). Part 2 sought to determine whether and how the participating organisation recognises consumers' contributions and to whom they offer remuneration. This was the largest part of the survey and incorporated 35 questions, including 10 multi‐choice and 25 free‐text questions. Our aim was to provide participants with an opportunity to share their experiences and perspectives. Part 3 focused on additional costs of consumer engagement, including but not limited to transport, parking, pre‐reading (i.e., reading materials related to meetings before attendance) and how research organisations remunerate these additional costs in practice. Part 4 explored the process of remuneration of participating organisations and any burdens encountered in practice. The last section, Part 5, enabled participants to share their opinions about remuneration guidelines: whether they thought it would be helpful to have national guidelines for remuneration and what they thought would make it easier for their remuneration practice regarding consumer engagement. Participants were not required to answer every question; we also utilised the skip and display logic in Qualtrics to enable participants to answer only questions relevant to them, thus improving their survey experience. As a result, the number of participants varied slightly between questions.

### 2.3 Data Analysis

We employed descriptive statistics to analyse multiple‐choice questions and content analysis to explore open‐text answers. Descriptive statistics were conducted using IBM SPSS statistics 29 to examine characteristics of participating research organisations, the prevalence of different targets and approaches for remuneration, whether formal processes were in place and guidelines for remuneration were followed, and whether participants believed it would be helpful to have national guidelines for remuneration. Simple frequencies were calculated in SPSS. Data from open‐text fields enabling participants to answer open‐ended survey questions and specify ‘other’ characteristics not described in the available multiple‐choice answer categories were imported into NVivo 14 (QSR International) qualitative data analysis software. Data were coded and synthesised into themes. Thematic analysis was employed to systematically code and synthesise the data, facilitating the identification of patterns and overarching themes within participants' responses. All quantitative and qualitative analyses were completed by one member of the research team (M.Z.), with regular review and discussion with other team members (J.D., A.P. and A.R.).

## 3 Results

The survey was open from 10 December 2023 to 29 February 2024. Of the 503 recipients who opened the email invitation, 124 accessed the survey via Qualtrics. Among these, 110 participants completed the survey (22% response). One possible reason for the low response rate is that the survey was conducted over the holiday season in Australia when many people take annual leave. Participant characteristics are presented in Table 1.

**Table 1:** Descriptive statistics of participating organisations (*N* = 110).

Variable	*n*	%
Type^a^		
University	52	47.3
Medical Research Institute	32	29.1
Research organisation	11	10.0
Healthcare provider	2	1.8
Non‐government organisation	9	8.2
None of the above	3	2.7
Type of non‐profit or for‐profit^a^		
Non‐profit	98	89.1
For‐profit	11	10.0
Size of organisations^a^		
> 50 employees	89	80.9
21–50 employees	6	5.5
11–20 employees	5	4.5
< 10 employees	9	8.2

^a^ *N* = 109, missing for one participant.

Most participants (86%) were from the academic field, with 47% from universities, 29% from medical research institutes and 10% from research organisations. Similarly, most participants were from not‐for‐profit organisations (89%) and larger organisations (81%) with > 50 employees. Only three participants selected ‘none of the above’ when asked about the type of organisation and nominated state government, research consultancy and community health representative when asked to describe their organisation.

### 3.1 Recognition of Consumer Engagement

A large proportion of participants (92%) supported consumers' contribution to health research; 56% of participants chose financial remuneration and 36% of participants chose non‐financial remuneration (Table 2). In relation to financial remuneration, 39% of participants used gift cards, 30% of participants paid directly to consumers' bank accounts, 18% of participants used prepaid visa or master cards and 6% of participants paid consumers cash. In relation to non‐financial recognition, 28% of participants recognised consumers' contribution academically, including co‐authorship of or acknowledgement on publications, 22% of participants provided training or skills development opportunities for consumers as recognition and 22% of participants organised team‐building activities to support consumers' contribution.

**Table 2:** Descriptive statistics for supporting consumer contribution.

Variable	*n*	%
Whether support consumer contribution		
Yes	101	91.8
No	3	2.7
Unsure	6	5.5
How to recognise contribution		
Financial remuneration	86	56.6
Non‐financial remuneration	55	36.2
None	11	7.2
How to recognise contribution financially*		
Payment via a gift card	71	39.9
Payment via prepaid Visa or Mastercard	32	18.0
Direct payment—into a bank account	54	30.3
Direct payment—cash	11	6.2
Other	10	5.6
How to recognise contribution non‐financially*		
Provision of training or opportunities for skills development	37	22.7
Academic recognition, such as co‐authorship or acknowledgement of publications	46	28.2
Co‐presentations at conferences	33	20.2
Team‐building activities, such as social gatherings	36	22.1
Other	11	6.7

* Multiple‐choice responses.

### 3.2 Financial Recognition of Consumer Engagement

#### 3.2.1 Targets for Financial Recognition

Financial remuneration was most commonly (24% of organisations) provided for consumers involved in advisory groups or steering committees and in research teams (Table 3). 21% of participating organisations remunerated consumers for participation in non‐treatment‐related research, such as interviews and focus groups, while it was less common to remunerate consumers for participation in treatment‐related studies (e.g., clinical trials) (13%) or consumer involvement coordinators (8%) and advocacy organisations (7%).

**Table 3:** Descriptive statistics for targets for consumer remuneration.

Variable	*n*	%
Who is the target for remuneration*		
Consumers involved in advisory groups or steering committees	73	24.4
Consumers involved in research teams, i.e., lived experienced researchers	72	24.4
Consumers participating in treatment‐related studies, e.g., clinical trials	41	13.7
Consumers participating in non‐treatment‐related research, e.g., interviews and focus groups.	64	21.4
Involvement coordinators who facilitate access to consumers (e.g., members of disease advocacy organisations)	25	8.4
Consumer‐led non‐government organisations advocating on behalf of consumers	20	6.7
Other	4	1.3
Total	299	100%

* Multiple‐choice responses.

#### 3.2.2 Methods of Financial Remuneration

The most common remuneration methods used by participating organisations were paying (1) an hourly rate or (2) an amount per meeting/activity (Table 4). This trend was similar across the five different roles consumers may adopt. It was particularly evident for consumers who were members of advisory groups or steering committees and those who were members of research teams, with 45% of participants using an hourly rate. In contrast, fewer participants reported using an hourly rate to recognise consumers' contributions for participating in research (14%), whereas 47% utilised a per meeting/activity rate as their remuneration method.

**Table 4:** Methods of financial remuneration for consumer engagement.

How to remunerate	Advisor*		Team member#		Participant**		Coordinator##		Representative***	
	*n*	%	*n*	%	*n*	%	*n*	%	*N*	%
Amount per hourly rate for involvement	31	44.9	41	48.2	6	14.3	8	32	4	20
Amount per meeting/activity	32	46.4	25	29.4	20	47.6	3	12	2	10
Ongoing retainer	0	0	5	5.9	1	2.4	4	16	2	10
No payment	1	1.4	4	4.7	4	9.5	2	8	2	10
Other	5	7.2	8	9.4	4	9.5	3	12	8	40
Unsure	0	0	2	2.4	7	16.7	5	20	2	10
In total	69	100%	85	100%	42	100%	25	100%	20	100%

* Advisor: Being a member of advisory groups or steering committees.

** Participant: Being a participant in research, including treatment‐related studies, e.g., clinical trials and non‐treatment‐related research, e.g., interviews and focus groups.

*** Representative: Being a representative for consumer‐led non‐government organisations advocating on behalf of consumers.

^#^ Team member: Being a member of a research team, i.e., consumers with lived experience.

^##^ Coordinator: Being a coordinator for consumer engagement who facilitates access to consumers (e.g., members of disease advocacy organisations).

### 3.3 The Process of Recognising Consumer Engagement

Participants were queried on several aspects of the recognition process for consumer engagement, including the presence of a formal onboarding process, provision of information on potential impacts of paid remuneration, allowance for consumers to decline financial remuneration and documentary requirements regarding remuneration. This survey segment aimed to explore the remuneration process for consumer engagement in current practice.

Equal numbers of participants (43.2%) had or did not have a formal process for onboarding consumers (Table 5‐1). 44% of participants indicated they provided information on the potential impacts of paid remuneration, while 29% and 26% of participants did not provide or were unsure about such information. Nearly 60% of participants allowed consumers to choose not to accept financial remuneration or to donate to a nominated charity.

**Table 5‐1:** Descriptive statistics for remuneration process of engagement.

	Formal process*		Impact information#		Not accept remuneration**		Documentary requirements##	
	*n*	%	*n*	%	*n*	%	*n*	%
Yes	35	43.2	15	44.1	20	58.8	28	82.4
No	35	43.2	10	29.4	6	17.6	4	11.8
Unsure	11	13.6	9	26.5	8	23.5	2	5.9
In total	81	100%	34	100%	34	100%	34	100%

*A formal process for onboarding consumers.

**Allowing consumers to choose not to accept financial remuneration or to donate to a nominated charity.

#Information of potential impacts of paid remuneration?

##Having documentary requirements related to remuneration.

In terms of potential burdens associated with onboarding consumers, such as documentary requirements, over 80% of participants reported having paperwork related to remuneration. 80% of participating organisations reported using 1–2 forms for onboarding consumers in the first instance and for each occasion of engagement (Tables 5‐2). Over half of the participants reported spending 1–5 h onboarding a consumer while spending less than 3 h organising remuneration for five consumers following a focus group or interviews, with over 70% of participants selecting this time frame.

**Table 5‐2:** Descriptive statistics for remuneration process of engagement.

Forms	First instance*		each occasion#			Onboard a consumer**		Remuneration for five consumers##	
	*n*	%	*n*	%	Hours	*n*	%	*N*	%
1–2	21	75	22	81.5	< 1	5	14.7	15	45.5
3–4	2	7.1	0	0	1–2	12	35.3	10	30.3
> 5	1	3.6	0	0	3–5	8	23.5	2	6.1
Unsure	4	14.3	5	18.5	6–8	2	5.9	0	0
					> 8	3	8.8	0	0
					Unsure	4	11.8	6	18.2
In total	28	100%	27	100%		34	100%	33	100%

* How many forms are in the first instance when onboarding consumers?

** How many hours to onboard a healthcare consumer?

^#^ How many forms for each occasion of engagement?

^##^ How many hours to organise remuneration for five consumers following a focus group or interviews?

### 3.4 Additional Costs of Consumer Engagement

Aside from payment to recognise consumers' contributions, there was also remuneration for the additional cost consumers incurred to participate.

As shown in Table 6, 93% of participants indicated that they remunerated consumers for the additional costs of being involved, the majority of which were transport (taxi or bus), parking fees and time for pre‐reading (total 87% of responses). Over 80% of organisations either provided taxi vouchers upfront or reimbursed costs afterwards. For parking fees, 15% of organisations paid upfront, and over 40% reimbursed parking fees afterwards, while 31% of organisations provided a parking permit. For the pre‐reading, over 85% of participating organisations incorporated this into the hourly rate for involvement.

### 3.5 Opinions About Remuneration Guidelines

#### 3.5.1 What Would Make It Easier?

Using an open‐response item, participants were asked what factors would make the remuneration process easier for them in practice. 67 out of 110 participants provided free‐text responses. The most frequently mentioned factors were the development of national guidelines (*n* = 24), the establishment of easy and supportive processes (*n* = 21) and the provision of funding support from relevant authorities (*n* = 15).

(a) National guidelines for remuneration

Specific reasons provided for the necessity of national guidelines included the importance of standardised rates and procedures and the various functions national guidelines could serve. Table 7 presents selected quotes from participants to illustrate these points.

**Table 6:** Descriptive statistics for additional costs of engagement.

Variable	*n*	%
Additional costs of engagement*		
Transport (taxi, bus)	49	32.0
Parking fees	46	30.1
Time for pre‐reading	39	25.5
Something else not listed above	12	7.8
No, we do not remunerate for additional costs such as the above	7	4.6
Unsure	0	0
Total	153	100%
How to remunerate for taxi fares?	*n*	%
Provide taxi voucher upfront	24	40.7
Reimburse taxi fare	24	40.7
Unsure	2	3.4
Other	9	15.3
Total	59	100%
How to remunerate for parking?	*n*	%
Provide parking permit	23	31.5
Pay for parking upfront	11	15.1
Reimburse for parking fees paid	32	43.8
Unsure	1	1.4
Other	6	8.2
Total	73	100%
How do you remunerate for pre‐reading?	*n*	%
Incorporate into an hourly rate for involvement	36	85.7
Set rate	4	9.5
No payment	1	2.4
Other	1	2.4
Total	42	100%

* Multiple‐choice responses.

(b) Easy and supportive remuneration process

Four themes were identified: simplified payment processes, reduced paperwork, the need for administrative support, and streamlined onboarding processes and systems. Table 7 provides detailed quotes from participants supporting these aspects.

(c) Funding support from authorities

Two key themes were identified: (1) the necessity of consistent funding support and (2) the proposal to establish non‐project‐specific funding streams. Several participants pointed to the importance of financial support from major authorities, such as the National Health and Medical Research Council (NHMRC) and the Medical Research Future Fund (MRFF), particularly in light of the current financial pressures on research budgets. Direct quotes illustrating these points are presented in Table 7.

#### 3.5.2 Whether It Would Be Helpful to Have National Guidelines or Recommendations for Consumer Remuneration?

As indicated in Table 8, over 93% of participating organisations expressed support for the development of national guidelines or recommendations for consumer remuneration, with only five participants expressing disagreement. When asked to elaborate on their reasons for supporting national guidelines, 69 participants provided at least one rationale through free‐text responses. The most frequently mentioned reasons included facilitating consistency across organisations, supporting research organisations, removing barriers to practice and motivating consumer engagement.

**Table 7:** Qualitative results on ‘what factors would make the remuneration process easier in practice?’.

Themes	Quotes
National guidelines for remuneration	
Necessity of national guidelines	‘…national guidance/education materials on hourly rates, and options for remuneration.’ ‘Having a consistent remuneration guideline across Australia in terms of the amount you provide and how you're paying that. This then needs to translate through the organisation to make the process straightforward.’
Importance of standardised rates and procedures	‘Standardised basic rates that are agreed upon by all research organisations,’ ‘Standard process across Australia, so everyone knows how to pay, and organisations won't be at risk.’
Various functions of national guidelines could serve	‘Guidelines or standards for payment to refer to would assist with budgeting for grant applications and justify requests for funding.’ ‘A national approach to consumer remuneration would be amazing and help drive consistency. All universities would be able to take this to their researchers, finance teams, write these rates in their grant applications, etc.’
Easy and supportive process for remuneration	
Simplified payment processes	‘We operate our accounts through a university and health district. The financial systems of both organisations are very complex and do not manage consumer payments easily, e.g., they require invoices to be issued for payment.’ ‘Smoother onboarding in the university system, easier payment process (currently is a spreadsheet system, should be integrated into our online system).’
Reduced paperwork	‘Administration often requires several hoops and lots of double‐checking to ensure payment is made,’ ‘Not requiring so much paperwork. I recently had to completely overhaul how I had been doing reimbursement for over a decade because the university decided not to accept it.’
Need for administrative support	‘It's easy but time‐consuming for researchers running multiple projects. It would help to have an administrative assistant for all of this, so researchers can focus on the content and academic documentation.’ ‘Coordinated support by a central area at the university—which sets and applies policy and procedures, and manages payments (like travel or claim expenses, which are much easier once you're set up).’
Streamlined onboarding process and systems	‘Onboarding processes can also be really burdensome and prevent people from wanting to get involved or create a not very diverse pool of people able to manage involvement.’ ‘Easier onboarding systems—it is so hard for some consumers to find things such as tax file numbers or to use the online forms and platforms to fill in their information.’
Funding support from authorities	
Necessity of consistent funding support	‘Funding from funding bodies like NHMRC and MRFF is essential.’ ‘Funding availability is crucial! We do not have funds earmarked for this purpose.’ ‘We already face a significant shortfall in research funding, and if we are also to remunerate consumers, what will be left for the actual research? The administering organisations and funding bodies need to account for this if it is to happen.’
Proposal to establish non‐project‐specific funding streams	‘Having a central fund within the organisation for consumer remuneration, rather than making it project‐specific, would be beneficial. Often, Consumer Reference Groups (CRGs) support multiple teams and projects, making it challenging to determine which team or project code should bear the remuneration costs.’

**Table 8:** Descriptive statistics for remuneration suggestions of engagement.

Variable	*n*	%
Is it helpful to have national guidelines or recommendations for remuneration?		
Yes	72	93.5
No	5	6.5
Total	77	100%

(a) Facilitating consistency across organisations

Twenty‐one participants endorsed the establishment of national guidelines to ensure consistency in remuneration across organisations. Four reasons emerged in support of this proposal: addressing confusion caused by inconsistent remuneration payments, setting clear expectations for both researchers and consumers, offering guidance both within and between organisations and fostering collaboration between consumers and research bodies. Table 9 provides direct quotes from participants illustrating these points.

**Table 9:** Qualitative results on ‘Reasons to have national guidelines or recommendations for consumer remuneration’.

Themes	Quotes
Facilitating consistency across organisations	
Confusion arising from inconsistent remuneration payments	‘Consumers contribute to research across many organisations, and the lack of consistency in remuneration payment creates confusion amongst consumers and researchers.’ ‘To ensure consistency across organisations, so consumers aren't expected to provide support, feedback, etc., for free from some services but are remunerated appropriately from others.’
Setting expectations for both researchers and consumers	‘Provides consistency and sets expectations for researchers and consumers alike,’ ‘So that all involved have the same expectations and admin can be properly organised to remunerate promptly. Having a consistent policy for all would make processes easier, fair, and equitable.’ ‘There should be consistency across all organisations regarding consumer engagement. Remuneration guidelines would ensure that consumers understand the “rules” of remuneration, which would not vary from place to place.’
Providing guidance within and across organisations	‘Having a policy or guideline from a reputable source will also make it easier to set up processes within organisations and to set expectations from both researchers and consumers around budgeting.’ ‘Guidance on standards for payment. Standardised amounts across projects set expectations for consumers and would be useful for justifying grant budgets.’
Promoting cooperation between consumers and research organisations	‘Having a national guideline would make it easier to promote the need for remuneration across the organisation,’ ‘National consistency in payments would allow organisations to follow best practice and consumers to know what to expect when working with different/multiple organisations.’
Supporting research organisations	
Enhance administrative efficiency	‘I personally think this would be useful and then the organisation would need to align with this. This would make it simpler to administer.’ ‘Having a clear national guide would be helpful and allow organisations to focus on specific issues that affect them.’
Support policy development	‘Having standardised rates is easier to create policy around and raise awareness.’ ‘It would allow a focus on specific needs and enable timely updates to policy.’
Assist in preparing ethics and grant applications	‘Guidelines can be useful to justify reimbursement in ethics applications, grant applications, and to other funders.’ ‘There is also the issue of finding funding to support consumer consultation in the early conceptualisation stages of a research project. Setting a national standard would support academic institutions to budget for such activities in the pre‐grant preparation stages with greater certainty.’ ‘Can budget for multisite, national studies with consumers involved across states.’
Removing barriers in practice	
Difficulties encountered without such guidelines	‘Different rates everywhere. Confusing for consumers. Institutions are also unsure if they're doing the right thing, so standardized guidelines would remove this concern.’
Problem of consumer representativeness	‘When consumers are not paid correctly, we will only attract consumers who can afford the time and expense of taking part and reduce the diversity of voices heard.’
Confusion among consumers and researchers	‘Consumers contribute to research across many organisations, and the lack of consistency in remuneration payments creates confusion amongst consumers and researchers. A standard process will remove one of the main barriers to consumers being paid in a fair and timely way.’
Variations across different jurisdictions.	‘The Commonwealth payment document is so complex you need a lawyer to interpret it! It's confusing and inconsistent. A set of National Rates and Guidelines would be helpful.’
Motivating consumers to be engaged	
Provide clarity and confidence to consumers	‘National guidance would not only support researchers in developing their budgets for grants, but also give some clarity and confidence to consumers when they engage in research.’
Empower consumers to advocate for fair remuneration	‘Many consumers are put off working with researchers through bad experiences where they feel they were taken advantage of (and likely were!). If there were guidelines on minimal remuneration for engagement, it would improve understanding of researchers that consumers need to be compensated, but also empower consumers to ask for what they deserve.’
Promote equal recognition for their value	‘Recognition of the valuable role consumers play in research,’ ‘For equity and to acknowledge consumer involvement appropriately.’

(b) Supporting research organisations

Nineteen participants highlighted the benefits of national guidelines in supporting research organisations. These benefits included improving administrative efficiency, aiding in policy development and providing assistance in the preparation of ethics and grant applications. Quotes supporting these views can be found in Table 9.

(c) Removing barriers to practice

Sixteen participants identified the removal of practical barriers as a reason for supporting national guidelines. These barriers included challenges faced in the absence of national guidelines, issues related to consumer representativeness, confusion among both consumers and researchers and discrepancies across different jurisdictions. Direct quotes from participants addressing these barriers are presented in Table 9.

(d) Motivating consumer engagement

Fifteen participants linked their support for national guidelines to the potential to motivate consumer engagement. They suggested that national guidelines would offer clarity and assurance to consumers, empower them to advocate for fair remuneration and promote equal recognition of their contributions. Supporting quotes from participants are available in Table 9.

## 4 Discussion

To our knowledge, this survey represents the most extensive examination of research organisations' perspectives on remuneration practices within the Australian context. The findings revealed that most research organisations advocate for the recognition of consumer contributions through both financial and non‐financial means. However, there is considerable variability in current remuneration practices and a lack of consensus on specific aspects, such as remuneration methods and rates. Notably, our findings suggest an overwhelming majority of participating organisations support the establishment of national guidelines or recommendations for consumer remuneration practices. Findings provide a comprehensive overview of current remuneration practices and may have significant implications for both individuals and the broader consumer engagement community.

Previous research indicates that recognising consumers' contribution is crucial for successful consumer engagement in health research [5, 22, 23]. Multiple studies have demonstrated that offering financial or non‐financial compensation plays a critical role in supporting sustained and active consumer engagement [24, 25, 26]. Recognition methods can be financial (e.g., honoraria) or non‐financial (e.g., co‐authorship). However, our research found that organisations employ a variety of methods for recognising and remunerating consumers' contributions, potentially presenting dilemmas for researchers or institutions who are seeking guidance in relation to this. For example, roughly equal proportions of participants indicated that they utilised academic recognition, such as co‐authorship of or acknowledgement on publications, provision of training or skills development opportunities, and employment of team‐building activities, such as social gatherings, to show their appreciation for consumers' contributions. These non‐financial recognition methods align with the reported practices in the literature, though the distribution percentages differ. Findings from a recent systematic review [10] on recognising patient partner contributions to health research showed that the most common methods of non‐financial compensation reported were informal acknowledgement of research outputs (65%) and co‐authorship (49%), while only 28% of participants in our survey supported non‐financial academic recognition methods. Furthermore, a scoping review [16] of available policies and guidelines on supporting consumers' contribution compensation indicated that the most commonly recommended methods of non‐financial compensation were offering training opportunities to consumer partners (40%) and facilitating participation in conferences (38%). These discrepancies in the non‐financial recognition of consumer's contributions may cause confusion in practice, potentially hindering consumer engagement in health research.

These inconsistencies are also evident in financial remuneration practice. Financial remuneration, in particular, can facilitate more equal and accessible engagement by removing significant barriers [27]. Without financial remuneration, only consumers with the time and resources are able to participate in research. A systematic review from 2023 identified four commonly reported methods of financial compensation: honoraria, gift cards, salary and scholarships [10]. Consistent with these findings, > 50% of participants in our study provided financial remuneration in recognition of consumers' contributions, with indirect payment methods such as gift cards or prepaid Visa/Mastercard being the most common. However, there are substantial differences in specific remuneration rates. For example, 48% of participants used a per meeting/activity rate to remunerate consumers involved as team members, while 29% preferred an hourly rate. Conversely, only 14% of participants used an hourly rate for consumers involved as research participants, with 47% opting for a per meeting/activity rate. While we were unable to examine the extent to which the level of engagement was associated with the payment methods reported, this aligns with recommendations from Australian state and territory remuneration guidance documents that suggest different compensation rates based on the roles of consumers in research [17, 18, 19, 20, 21] and presents an opportunity for future research to do so. These policy documents vary in their recommendations, and while it is reasonable for different states or territories to develop unique remuneration guidelines tailored to their local context, having multiple guidance documents can pose challenges.

Similarly, the recognition process of consumer engagement in the current Australian context varied substantially between organisations, with less than half of the responding organisations reporting a formal process for remuneration of consumer engagement. The implications of this were reflected in the free‐text responses indicating that clear and consistent guidelines to inform remuneration practice would be helpful. This finding aligns with barriers identified in previous literature [4, 5, 10, 28, 29], which emphasises the important role of institutional policies and guidance [10]. These barriers can limit a research team's ability to offer financial remuneration to consumer partners. Two other notable differences highlighted in the survey responses related to organisations' provision of information about the potential impacts of paid remuneration and whether consumers are given the option to decline financial remuneration. The potential impacts of financial remuneration on consumers include jeopardising disability or social security payments or affecting income tax rates. This impact is also reported in the literature [7, 10] as an important consideration, acknowledging that consumers can refuse remuneration and the need for researchers and consumer partners to co‐develop a remuneration strategy that balances recognition with potential risks associated with financial remuneration [10].

The administrative burden associated with the remuneration process reported in our study was reflected in reports of substantial documentary requirements and time, with the majority of participants reporting that onboarding a consumer took more than 1 h. This significant time investment, particularly if a research project involves many consumers, generates an additional bureaucratic burden for researchers. This has been echoed in a US study indicating that funding recipients spent 42% of their time on administrative activities, including pre‐ and post‐award processes [30]. Alleviating the administrative burden of the process of consumer engagement would be a motivating factor for researchers to engage consumers in their research and again presents a potential challenge to be tackled by future research.

Additional costs, also considered eligible for reimbursement for consumer engagement, refer to the expenses associated with consumers' participation in research projects [31]. These costs could include transport, parking fees, time for pre‐reading, accommodations, conference attendance, babysitting/caregiver services, home printing, technology or internet access and so forth [6, 16]. Our survey results indicated significant variability in responses. Approximately one‐third of participants supported payment of transport, parking fees and time for pre‐reading, though the methods for remunerating these costs varied across organisations. Regarding transport and parking remuneration, 40% of participants answered that their organisation provides support upfront (e.g., taxi voucher or parking permit), while 40% offered reimbursement after payment. This can create a dilemma for consumers who must initially cover these costs themselves and later claim reimbursement, potentially navigating complex billing systems or providing invoices [6]. Such inconsistency in current reimbursement practices may pose challenges for both researchers and consumers new to engagement in health research.

Notably, an overwhelming majority of participants (92%) in our study supported recognition of consumers' contributions to health research, employing both financial and non‐financial approaches. Similarly, the majority supported the establishment of national guidelines or recommendations for consumer remuneration practices. A scoping review of policies and guidelines supporting patient partner compensation practices in April 2022 did not identify any national documents from Australia [16]. This highlights that there are currently no national guidelines on remuneration of consumer engagement in Australia. As mentioned, guidelines exist across individual Australian states and territories [17, 18, 19, 20, 21]; achieving consistency in these would enhance consumer recognition practices and facilitate broader consumer engagement in health research.

## 5 Strength and Limitations

This project was conducted in collaboration with Research Australia, involving active engagement from eight of its member organisations within a working group. One of the project team members, who is also part of this working group, played an instrumental role in our research and is included as a co‐author in this publication. A key strength of this paper is the formation of the working group, whose expertise and support guided the study. The co‐development of the survey exemplifies this collaboration and ensures the construct validity of the questions asked. Another strength is the pilot testing of the survey by three lived‐experience researchers. Their expertise in research ensured the survey's content validity, while their experiential knowledge from lived experience guaranteed its applicability.

However, several limitations must be noted. First, the study is limited to the views of participating organisations that are members of Research Australia. While these member organisations are distributed across Australia and may reflect the perspectives of a broader research community, the study may have missed voices from research organisations that do not belong to this formal network. Additionally, without an existing portfolio for the member organisations of Research Australia, we could not determine whether the responding organisations accurately represent the distribution of large, medium and small research organisations in Australia. Both these factors potentially impact the generalisability of the findings. While the working group included two consumer‐led research organisations, the voices of consumers themselves were largely not included in the research. Future research focusing on the development of consumer recognition and remuneration guidelines must include the consumer perspective as an equal voice in developing these. Lastly, the anonymity of the survey means we cannot verify if multiple responses came from the same organisation, potentially affecting the heterogeneity and representativeness of the survey results.

### 5.1 Implications for Future Research

This study provides an excellent foundation for future studies exploring appropriate models to support the recognition of consumers' contributions to health research. While this study was largely informed by the voice of health research organisations, future models need to be co‐produced with consumers, health research organisations, disease advocacy organisations and health services. This is critical to ensure that they encompass a variety of needs and capacities, including the availability of funding to support the financial remuneration of consumers.

## 6 Conclusion

This study provides a comprehensive overview of the current recognition and remuneration practices of consumer engagement in health research in Australia. The responses indicate that most research organisations support recognising consumer contributions through both financial and non‐financial means. However, the variability in current practices and the lack of consensus on specific aspects, such as remuneration rates and methods, underscore the need for national guidelines. Development of such guidelines in collaboration with research organisations and consumers is an essential next step to optimising consumer engagement in Australian research.

Statements relating to our ethics and integrity policies: data availability statement, funding statement, conflict of interest disclosure, ethics approval statement and patient consent statement.

## Author Contributions

**Mingming Zhou:** conceptualisation, methodology, writing – original draft, writing – review and editing, software, data curation, investigation, validation, formal analysis, visualisation, project administration. **Julia Dray:** supervision, conceptualisation, methodology, data curation, formal analysis, writing – review and editing. **Anne Parkinson:** conceptualisation, methodology, data curation, supervision, formal analysis, validation, writing – review and editing, investigation. **Alice Richardson:** formal analysis, writing – review and editing, supervision, methodology. **Lucy Clynes:** conceptualisation, data curation, writing – review and editing, resources. **Jane Desborough:** conceptualisation, methodology, data curation, supervision, formal analysis, validation, writing – review and editing, resources, investigation.

## Ethics Statement

This study received ethics approval from the Australian National University Human Research Ethics Committee (Protocol 2023/1263). All participants provided informed consent.

## Conflicts of Interest

The authors declare no conflicts of interest.

## Supporting information

Appendix 1 Final 0328.

## Data Availability

The data that support the findings of this study may be available from the corresponding authors upon reasonable request.
