# Supplementary material for: Consumers' Contribution to Health Research: Australian Research Organisations' Perspectives
Source: Health Expect. 2025 Apr 28;28(3):e70258. doi: 10.1111/hex.70258 (PMC12037703; doi:10.1111/hex.70258)
Supplement: Supplementary file 1 — Appendix 1 Final 0328. [file HEX-28-e70258-s001.docx]

**Appendix 1**

**Survey in Qualtrics**

**Block 1 cover**

You have been invited to participate in this survey as your organisation is a member of Research Australia.

This brief survey is about your experiences and approaches to recognising consumers for their contributions to research, including financial remuneration, within your work at this organisation.

The ethical aspects of this research have been approved by the ANU Human Research Ethics Committee (Protocol 2023/1263).

Please read the [Participant Information Sheet](https://anu.au1.qualtrics.com/CP/File.php?F=F_5gxCn2YSlSKKGfs). You may like to download and save a copy for your records.

Completion of this survey indicates that you have read and understood the participant information sheet provided, and consent to participate in this study.

| **Topic** | **Questions** |
| --- | --- |
| **Block 2 basics**  1. About your organisation | 1. What type of organisation do you work for (please select one):   1. University 2. Medical Research Institute 3. Pharmaceutical company 4. Medical technology company   e) Research organisation  f) Health care provider  g) Non-government organisation  j) None of the above (go to Q2)  2. Please describe the type of your organisation: _____________  3. Is your organisation (please select one):  a) For-profit  b) Not for profit  4. How many employees are in your organisation?  a) < 10 employees  b) 11 – 20 employees  c) 21 – 50 employees  d) >50 employees |
| **Block 3 recognition**  2. Recognition of consumer engagement | 5. Does your organisation support health care consumers to contribute to research?  a) Yes (go to Q6)  b) No (skip to the end of survey)  c) Unsure (go to Q6)  6. How does your organisation recognise these contributions from health care consumers? (select all that apply)  a) Financial remuneration (go to Q7, and then Q40)  b) Non-financial remuneration (go to Q9)  c) None (skip to Q50)  7. How does your organisation recognise these contributions? (Select all that apply)  a) Direct payment - into a bank account (go to Q40)  b) Direct payment – cash (go to Q40)  c) Payment via a gift card (e.g., Coles, Woolworths)  d) Payment via prepaid Visa or Mastercard  e) Other (go to Q8)  8. Please describe the other type of financial remuneration in your organisation not listed:  ________________________  9. How does your organisation recognise these contributions non-financially? (select all that apply)  a) Provision of training or opportunities for skills development  b) Academic recognition, such as co-authorship or acknowledgment on publications  c) Co-presentations at conferences  d) Team building activities, such as social gatherings  e) Other (go to Q10)  10. Please describe the other type of non-financial remuneration in your organisation not listed: __________________________________ |
|  | 11. Who does your organisation remunerate? (Select all that apply)   1. Consumers involved in advisory groups or steering committees (go to Q15) 2. Consumers involved in research teams i.e., Lived experienced researchers* (go to Q20) 3. Consumers participating in treatment related studies e.g., clinical trials. (go to Q25) 4. Consumers participating in non-treatment related research e.g., interviews, focus groups. (go to Q25) 5. Involvement coordinators who facilitate access to consumers (e.g., members of disease advocacy organisations) (go to Q30) 6. Consumer-led non-government organisations advocating on behalf of consumers (go to Q 35)   g) Other (go to Q12)  12. Please describe who else your organisation provides remuneration to apart from those listed: __________________________________  (If the text response is not empty, then will go to Q13)  ***Lived experience researcher** ^[5]^ refers to a person who draws on their lived experience of disability, mental health, or any other health conditions to inform the research.  13. How does your organisation remunerate this group (e.g. hourly rate, per meeting, ongoing retainer, unsure)? __________________________________  (If the text response is not empty, then will go to Q14)  14. How much does your organisation pay for this type of remuneration?  15. How does your organisation remunerate/reimburse consumers for being a member of advisory groups or steering committee?  a) Amount per hourly rate for involvement (go to Q17)  b) Amount per meeting/activity (go to Q18)  c) Ongoing retainer (go to Q19)  d) No payment  e) Other (go to Q16)  16. Please describe the other type of remuneration for being a member of advisory groups or steering committee in your organisation not listed:  17. How much do you pay consumers per hourly rate for involvement?  18. How much do you pay consumers per meeting/activity for involvement?  19. How much do you pay consumers as an ongoing retainer for involvement?  20. How does your organisation remunerate/reimburse consumers for being a member of a research team?  a) An hourly rate for involvement (go to Q22­)  b) Amount per meeting/activity (go to Q23)  c) Ongoing retainer (go to Q24)  d) No payment  e) Other (go to Q21)  f) Unsure  21. Please describe the other type of remuneration for being a member of research team in your organisation not listed:  22. How much do you pay consumers for being a member of a research team per hour?  23. How much do you pay consumers for being a member of a research team per meeting/activity?  24. How much do you pay consumers as an ongoing retainer?  25. How does your organisation remunerate/reimburse consumers for being a participant of research, such as clinical trials, interview/focus group?  a) An hourly rate for involvement (go to Q 27)  b) Amount per meeting/activity (go to Q 28)  c) Ongoing retainer (go to Q29)  d) No payment  e) Other (go to Q26)  f) Unsure  26. Please describe other type of remuneration for being a participant of a research in your organisation not listed:  __________________________________  27. How much do you pay consumers for being a participant of research per hour?  __________________________________  28. How much do you pay consumers for being a participant of research per meeting/activity?  29. How much do you pay consumers for being a participant of research as ongoing retainer?  __________________________________  30. How does your organisation remunerate/reimburse consumers for being a coordinator who facilitates access to consumers (e.g., members of disease advocacy organisations)?  a) An hourly rate for involvement (go to Q32)  b) Amount per meeting/activity (go to Q33)  c) Ongoing retainer (go to Q34)  d) Other (go to Q31)  e) Unsure  31. Please describe the other type of remuneration for being a coordinator in your organisation not listed:  __________________________________  32. How much do you pay consumers for being a coordinator per meeting/activity?  __________________________________  33. How much do you pay consumers for being a coordinator per meeting/activity?  __________________________________  34. How much do you pay consumers for being a coordinator as an ongoing retainer?  __________________________________  35. How does your organisation remunerate/reimburse consumers for being a representative for consumer-led non-government organisations on behalf of consumers?  a) An hourly rate for involvement (go to Q37)  b) Amount per meeting/activity (go to Q38)  c) Ongoing retainer (go to Q39)  d) No payment  e) Other (go to Q36)  f) Unsure  36. Please describe the other type of remuneration not listed that you pay consumers for being a representative for consumer-led non-government organisations:  __________________________________  37. How much do you pay consumers for being a representative for consumer-led non-government organisations per hour?  __________________________________  38. How much do you pay consumers for being a representative for consumer-led non-government organisations per meeting/activity?  __________________________________  39. How much do you pay consumers for being a representative for consumer-led non-government organisations as an ongoing retainer?  __________________________________ |
| 3. Remuneration for additional costs of engagement | 40. Does your organisation remunerate or reimburse consumers for the following additional costs they may incur in order to be involved in research? (Select all that apply)  a) Transport (taxi, bus) (go to Q42, then go to Q49)  b) Parking fees (go to Q44, then go to Q49)  c) Time for pre-reading (go to Q46, then go to Q49)  d) Something else not listed above (go to Q41)  e) No, we do not remunerate for additional costs such as the above.  f) Unsure  41. Please describe the additional costs not listed that your organisation remunerates consumers for:  __________________________________ |
| 4. Mode of payment for additional costs of engagement | 42. How does your organisation remunerate or reimburse consumers for taxi fares?  a) Provide taxi voucher up-front  b) Reimburse taxi fare  c) Unsure  d) Other (go to Q43)  43. Please describe other ways not listed in previous options that your organisation remunerates or reimburse consumers for text fares:  44. How does your organisation remunerate or reimburse consumers for parking?  a) Provide parking permit  b) Pay for parking upfront  c) Reimburse for parking fees paid  d) Unsure  e) Other (go to Q45)  45. Please describe other ways not listed in previous options that your organisation remunerates or reimburse consumers for parking:  _________________________________  46. How does your organisation remunerate or reimburse consumers for pre-reading?  a) Incorporate into hourly rate for involvement  b) Set rate (go to Q47)  c) No payment  d) Other (go to Q48)  e) Unsure  47. Please describe the set rate when your organisation remunerates or reimburse consumers for pre-reading:  _________________________________  48. Please describe other ways not listed when your organisation remunerates or reimburse consumers for pre-reading:  _________________________________  49. Please describe if there is something else not listed when your organisation remunerates or reimburse consumers for taxi fares, parking and pre-reading?  _________________________________ |
| **Block 4 process**  5. Process of remuneration and potential burden | 50. Does your organisation have a formal process for onboarding consumers into research projects? For example, induction, training, or other support. Please describe:  a) Yes (go to Q51, then go to Q52)  b) No (go to Q63)  c) Unsure (go to Q63)  51. What is the formal process for onboarding consumers into research projects in your organisation? For example, induction, training, or other support.  _________________________________  52. If the remuneration rates of your organisation are publicly available, please insert the link here:  _________________________________  53. Does your organisation provide information to consumers of potential impacts of paid remuneration for involvement in research on their personal situation (such as impact on social security payments)?  a) Yes b) No c) Unsure  54. Does your organisation allow consumers to choose not to accept financial remuneration or to donate these funds to a charity of their choice?  a) Yes b) No c) Unsure  55. Does your organisation have documentary requirements related to remuneration, such as the requirement for consumers to complete specific forms?  a) Yes (go to Q56)  b) No (go to Q63)  c) Unsure (go to Q63)  56. How many forms in the first instance:   - - 1. 1-2; b) 3-4; c) >5; d) Unsure   57. How many forms for each occasion of engagement:   1. 1-2; b) 3-4; c) >5; d) Unsure   58. Who is responsible for managing the process of onboarding and remuneration of consumers? _____________  59. How many hours does it take your organisation to onboard a health care consumer? (Excluding training for consumers)  a) <1; b) 1-2; c) 3-5; d) 6-8; e) >8; f) Unsure  60. How many hours does it take to organise remuneration for five consumers who are already registered in your system, following for example a focus group or series of interviews?  a) <1; b) 1-2; c) 3-5; d) 6-8; e) >8; f) Unsure |
| 6. Consumer engagement remuneration guidelines/ advice | 61. Do you follow any specific guidelines or advice for remuneration?   1. Yes (go to Q62) 2. No 3. Unsure   62. Please describe the specific guidelines or advice for remuneration your organisation follows:  ___________________________________________________________ |
| **Block 5 future**  7. Future suggestions | 63. What would make it easier to remunerate consumers who engage in research in your organisation? ________________________________________________________________  64. Does your organisation think it would be helpful to have national guidelines or recommendations for remuneration of consumers engaging in research?   1. Yes (go to Q65) 2. No (go to Q66)   65. Please explain your reason for choosing Yes __________________________________________________________  66. Please explain your reason for choosing No __________________________________________________________  67. To help us reach a broad range of people, please suggest any workplaces, organisations, research or consumer groups or networks you think we should contact to be a part of our research about consumer engagement:  _____________________________________________ |

Thank you for taking the time to complete this survey. We truly value the information you have provided. Your responses will contribute to our analyses of consumer remuneration in health research and may provide new clues for recognition of consumer engagement.

**References:**

1. Australian Research Council (ARC) (2020). ARC Medical Research Policy Version 2020.1, Australian Research Council, downloaded from <http://www.arc.gov.au?about-arc/program-policies/medical-research-policy>

2. Health Consumers NSW (2020). Health Consumers NSW, downloaded from <https://www.hcnsw.org.au/consumers-toolkit/who-is-a-health-consumer-and-other-definitions>

3. Gregory, J., “Conceptualising consumer engagement: A review of the literature”, Australian Institute of Health Policy Studies, Melbourne, Victoria, 2007.

4. National Health and Medical Research Council and Consumers Health Forum of Australia (2016). Statement on Consumer and Community Involvement In Health And Medical Research (Issue September), downloaded from <https://www.nhmrc.gov.au/about-us/publications/statement-consumer-and-community-involvement-health-and-medical-research>

5. Sydney Health Partners. Language Guide for Consumer and Community Involvement, downloaded from https://cdn.sydneyhealthpartners.org.au/wp-content/uploads/2023/03/CCI-Language-Guide.pdf.

6. Novak-Pavlic, Monika, et al. "Patients and Families as Partners in Patient-Oriented Research: How Should They Be Compensated?" *Journal of Patient-Centered Research and Reviews* 10.2 (2023): 82.

7. Fox, Grace, et al. "Recognizing patient partner contributions to health research: a mixed methods research protocol." *Research Involvement and Engagement* 8.1 (2022): 24.

8. Vargas, Carmen, et al. "Co-creation, co-design, co-production for public health: a perspective on definition and distinctions." Public health research & practice 32.2 (2022).

9. Dickert N, Grady C. What’s the price of a research subject? Approaches to payment for research participation. *N Engl J Med.* 1999; 341:198-203.
